# A Safety Evaluation of the Resumption of Elective Orthopaedic Services in Higher Risk Patients During the COVID-19 Pandemic

**DOI:** 10.7759/cureus.22339

**Published:** 2022-02-17

**Authors:** Joseph Dixon, Negin Mirtorabi, Joseph Cutteridge, Monil Karia, Thomas Pollard

**Affiliations:** 1 Trauma and Orthopaedics, Nuffield Orthopaedic Centre, Oxford, GBR; 2 Trauma and Orthopaedics, University of Oxford, Oxford, GBR; 3 Trauma and Orthopaedics, Royal Berkshire Hospital, Reading, GBR

**Keywords:** elective orthopaedic surgery, covid-19, total hip replacement (thr), green pathway, patient safety

## Abstract

Introduction

The COVID-19 pandemic has caused mass disruption to all aspects of society, with elective orthopaedics not spared. The pandemic has the potential to cause a tsunami of health burden in the community if elective services are not resumed to pre-pandemic levels of activity. Studies have shown that elective orthopaedics can be safely carried out in a COVID-19 free hospital. This study reviewed the transition in operating at an independent COVID-19 free hospital to an NHS hospital concurrently treating patients with COVID-19.

Methods

A strategy of phased relaxation of clinical comorbidity criteria was followed. Patients from the orthopaedic waiting list were selected according to these criteria and observed recommended preoperative isolation protocols. Operations were undertaken in the independent sector under the COVID-19 contract and the NHS site. Patients were assessed from all phases in the resumption of services. In-hospital and post-operative complications with specific enquiries regarding the development of COVID-19 symptoms or the need and outcome for COVID-19 testing at 14 days and six weeks was recorded.

Results

This study included 263 patients, of which 155 were female. The mean age of patients was 52.45. The mean BMI of all patients was 29.1 kg/m2. Additionally, 124 patients were American Society of Anesthesiologists (ASA) grade 1, 117 ASA grade 2 and 22 ASA grade 3 and 167 patients underwent a major operation, with total hip replacement being the most common operation. There were no in-hospital complications. No patients had a positive test result or symptoms of COVID-19 in the six-week post-operative period.

Conclusion

In summary, we demonstrated that elective orthopaedic surgery can be safely undertaken via a green pathway in a higher risk patient cohort when COVID-19 is prevalent in the community.

## Introduction

The SARS-CoV-2 (COVID-19) pandemic caused mass disruption to all aspects of society, with elective orthopaedics not spared. An estimated 516,000 planned operations were cancelled in the first wave of the pandemic in the United Kingdom (UK) [1]. Orthopaedic departments in the UK currently face a challenging situation with significant staff absences due to high numbers of positive COVID-19 cases set against a background of vastly expanded waiting lists [2-5]. Figures from the British Orthopaedic Association (BOA) show the total waiting list for trauma and orthopaedics in England now stands over 704,000 patients, with over 63,000 patients waiting over one year to begin treatment [4]. These figures have increased significantly over the last 12 months with a 30% increase in the number of patients waiting over two years for an operation. Orthopaedics had the second-lowest return to the expected capacity of inpatient planned care [6]. Another barrier to resuming elective care are patient concerns with regards to undergoing surgery during a pandemic with studies showing only 56% of patients wished to proceed with planned orthopaedic care [7]. These figures were mirrored in our department when patients were initially contacted at the onset of the pandemic [8]. However, deferring the burden of chronic musculoskeletal conditions will not make it go away. Many individuals awaiting joint replacement surgery have a severely reduced quality of life, enduring significant amounts of pain and suffering, described by some patients as a state 'worse than death' [9].

A previous study from our trauma and orthopaedics department at Royal Berkshire NHS Foundation Trust showed that elective orthopaedic services can be safely resumed in the independent sector at a time when COVID-19 is prevalent in the community with high levels of patient satisfaction with no COVID-19 related complications [8]. The previously published study was limited to a low-risk patient cohort, with all patients being American Society of Anesthesiologists (ASA) grade 1, indicating an otherwise healthy individual (8). The patient cohort for the previously published work was selected as they were deemed to be at the lowest risk of developing significant complications of COVID-19 post-operatively [10]. Operations were undertaken at designated green hospital sites (COVID-19 free) in the independent sector. Herein we review the transition period from operating in the Independent sector to elective orthopaedic operating at the National Health Service (NHS) District General Hospital (DGH) site. We will also assess the outcomes of patients with higher ASA grades.

## Materials and methods

Elective surgery at our trust was paused from the 17th March 2020 to allow our theatres to be converted into an intensive care facility in preparation for the surge in COVID-19 admissions. Elective surgery was reintroduced on 4th May 2020 in the independent sector with strict clinical criteria. Phase 1 documented the first 100 patients to undergo surgery under the new COVID-19 precautions. Patients were prospectively monitored for symptoms of COVID-19 or post-operative complications at 14 days. None of the first 100 patients required re-admission to the hospital for any complication. A single patient tested positive for COVID-19. The results from phase 1 have been published and demonstrated a safe process with high levels of patient satisfaction with the process [8]. Therefore, it was judged that the protocol was safe to progress to phases 2,3 and 4 of the re-start of elective orthopaedic procedures, with each successive phase reducing patient exclusion criteria (Table 1).

The four principles of staff, space, stuff and systems were considered throughout the process of re-starting to ensure the safety of our patients and staff. The clinical criteria for patient selection were based on the best available evidence at the time and were reviewed throughout [10-13]. For phases 1-4, the pre-operative patient and household isolation period was set at 14 days; however, this was later reduced to three days on the 3rd August 2020 for lower-risk patients as per instructions from Public Health England. For low-risk patients in phase 4, the isolation period was optional. Any patient with a positive SARS-CoV-2 RT-PCR test preoperatively or symptoms of COVID-19 had their operation postponed. Elective operating returned to our NHS DGH site on 3rd August 2020 (phase 4). The designated green pathways as implemented in the independent sector were followed. Patients were admitted to ring-fenced green zone beds and were operated on in designated green zone theatres. Staff would be posted in the green zone for the entirety of the working day to limit transmission.

Table 1 shows the phased clinical criteria for elective orthopaedic surgery.

**Table 1:** Phased clinical criteria for elective orthopaedic surgery * Routine risk assessment as would happen in normal circumstances when considering a patient for elective surgery in a private hospital with no high-dependency unit (HDU) facilities ** Moderate or severe asthma defined by daily use of steroid inhaler, or oral steroids or hospital admission within last 12 months *** Uncontrolled hypertension diagnosed as >140/90mmHg in community, >160/100mmHg in clinic, arrhythmia permitted provided controlled at preoperative assessment **** Meet all other comorbidities criteria

	Start Date	Age (years)	BMI (kg/m^2^)	ASA score	Co-morbidities permitted	Healthcare worker with patient contact within last 4 months	Vulnerable as defined by current NHS criteria	Number of Patients
Phase 1	4^th^ May 2020	<70	<30	I-II	None	No	No	100
Phase 2	15^th^ June 2020	<70	<40	I-II	Current smoker, mild asthma**	Permitted with isolation criteria	No	54
Phase 3	29^th^ June 2020	All ages if the clinical frailty score was 1-3	<40	I-II	Current smoker, mild asthma**, hypertension and/or arrhythmia (provided controlled) ***, CVA or TIA (unless within last year)	Permitted with isolation criteria	Yes, if only classified as such on age and meeting other criteria****	90
Phase 4	3^rd^ Aug 2020	Routine risk assessment*	Routine risk assessment*	Routine risk assessment*	Routine risk assessment*	Permitted with isolation criteria	Yes, with routine risk assessment*	119

Preoperative assessment and isolation requirements

Patients identified as eligible at each phase entered the preoperative assessment pathway previously published [8]. Patients were isolated along with their household prior to their surgical date, although household contacts were allowed to make essential journeys provided they followed government guidance at that time. Five days prior to surgery patients underwent preoperative assessment, including routine methicillin-sensitive *Staphylococcus aureus* (MSSA) swabbing for those undergoing prosthetic surgery, and screening questions related to COVID-19 symptoms. All patients had a SARS-CoV-2 RT-PCR test (nasopharyngeal and throat swab) 48 to 72 hours preoperatively and were provided with an information leaflet explaining the measures taken by the hospital to protect them from contracting COVID-19 [14].

On arrival at the hospital, all patients had their temperature checked and were screened for infection with a questionnaire. Patients were anaesthetised and extubated in theatre with just the anaesthetist and operating department practitioner (ODP) present. After the patient left the theatre, there was a five-minute delay prior to cleaning, to allow sufficient air changes. Upon discharge, patients were advised to isolate as per Public Health England recommendations. At the onset of the study, periodic regular staff testing had not been introduced; however, this was introduced from August 2020 with weekly PCR tests. Staff were advised to follow national guidance if they developed symptoms or tested positive. 

Post-operative assessment

All patients undergoing elective surgery were surveyed via telephone at 14 days and followed up within six weeks post-surgery. The period of 14 days was selected as this reflected the period in which a patient who had acquired COVID-19 through nosocomial transmission would have been expected to develop symptoms. The six-week period was selected as representing a period of potential post-surgical immunosuppression with a potential higher risk of community-acquired COVID-19 infection. All patients underwent surgery between 4 May and 14 August 2020. The study was registered locally as a service evaluation.

## Results

In this study, there were a total of 263 patients, of which 155 were female and 108 were male. The mean age of patients was 52.45 years; 136 patients were operated on at Circle Reading Hospital, 27 patients at the Berkshire Independent Hospital and 100 patients at the Royal Berkshire Hospital. All patients went through the COVID-19 protocol as set out above, with negative swab results at 48-72 hours pre-operatively. The mean BMI of all patients included in the study was 29.1 kg/m2, and 104 of the 263 patients had a BMI greater than 30; 124 patients were ASA grade 1, 117 ASA grade 2 and 22 ASA grade 3 and 167 patients underwent a major operation with total hip replacement being the most common major operation (Table 2).

**Table 2:** Table of operations by classification

Classification	n	Operation	n
Major	167	Total hip replacement	48
		Hip arthroscopy	22
		Total knee replacement	22
		ACL reconstruction	13
		Shoulder arthroscopic procedure	12
		Unicompartmental knee replacement	12
		Spinal discectomy/microdiscectomy	11
		Osteotomy	8
		Spinal decompression	8
		Trapeziectomy	3
		Revision of total hip replacement	1
		Total shoulder replacement	1
		Ankle arthrodesis	1
		Synovectomy MCPJ	1
		Calcaneocuboid fusion	1
		Reconstruction of ulnar ligament	1
		Acute ACJ stabilisation with Lars ligament	1
		Stabilisation of DRUJ	1
Intermediate	39		
		Knee arthroscopy	22
		Other hand and wrist procedure	9
		De Quervain’s release	2
		Scarf osteotomy	2
		Ankle arthroscopy	1
		Ankle debridement	1
		Stainsby's correction of lesser toe	1
		Toe amputation	1
Minor	57		
		Carpal tunnel decompression	16
		Hip joint injection	13
		Removal of metalwork	13
		Spinal injection minor soft tissue procedure	5
		Spinal injection	4
		Hip arthrogram	3
		Manipulation under anaesthesia of joint	3

Clinical assessment

There were no in-hospital complications. No patients had a positive test result or symptoms of COVID-19 in the six-week post-operative period. A patient from phase 4 had to return to the hospital for investigation of a DVT which was negative, and one patient re-presented to the hospital following a seizure. None of these patients had symptoms of COVID-19 and all were discharged without a test.

No patients developed symptoms of COVID-19 or received a positive test result during the transition period. Patients were also asked if any family members had developed COVID-19 symptoms during the post-operative period, in order to screen for transmission from asymptomatic carriers. The was no difference in risk of complication or developing symptomatic COVID-19 infection for patients operated in the Independent sector or NHS hospital site. 

This study included a higher risk cohort of patients who are more suspectable to severe disease and death from COVID-19 [15]. None of these patients (ASA grade 3) developed signs or symptoms of COVID-19 or had a positive test result. If these patients had contracted COVID-19 in hospital, none of them became symptomatic enough to require medical treatment or re-admission to hospital.

## Discussion

Discussion

This study has shown that major orthopaedic operations can be performed safely in an NHS DGH during a period in which COVID-19 is prevalent in the community, with low levels of complications. Patients classified in higher ASA grades, who are thought to be at higher risk of developing COVID-19 complications post-operatively, were protected from infection and recovered well [15-17]. Our trust was able to achieve satisfactory patient outcomes in the six-week post-operative period with no patients readmitted to the hospital within the study period.

Studies have estimated that the probability of an asymptomatic patient with SARS-CoV-2 receiving a false negative preoperative test was 0.07% (around 1 in 1,400) [18]. The risk of a patient with an undetected infection being admitted for surgery and subsequently dying from the COVID-19 disease is estimated at approximately 1 in 7000 [18]. This information and the results of this study should re-assure patients that elective orthopaedic surgery can be safely provided via a green pathway at an NHS site. 

This study has highlighted that Royal Berkshire NHS Foundation Trust was able to safely transition to elective operating at the main NHS DGH without resulting in adverse patient outcomes during this transition. There was a low transmission rate of COVID-19 in this study, with no positive test results from phase 2 of the resumptions of elective services.

There was no increase in transmission or complication rate for patients who were operated on in the COVID-19 free Independent sector or at the NHS site which was concurrently accepting patients with COVID-19. This should provide reassurance to patients and clinicians alike, especially in light of previous reports published at the outset of the pandemic, which demonstrated very high complication rates in patients undergoing operations when diagnosed with COVID-19 [17]. 

The UK vaccination programme is has made excellent progress with 84% of the population over 12 years old having had two doses of the vaccine [19]. COVID-19 however remains prevalent in the community with novel variants frequently arising. The protection that the vaccines will provide against new variants is unknown.

The authors acknowledge limitations to this study, patients were not routinely tested for COVID-19 in the immediate post-operative period, so there is a possibility that some acquired asymptomatic COVID-19 infection in the community.

A second limitation of this study was, in phase 2 and 3, the continued requirement of the independent sector to provide a green site to operate on COVID-19 negative patients. Having a designated COVID-19 free site is unlikely to be a practical solution for many departments.

## Conclusions

In summary, our trust has demonstrated that elective orthopaedic surgery can be safely undertaken via a green pathway when COVID-19 is prevalent in the community. This study has shown no greater risk of re-admission in patients with higher ASA grades which should provide reassurance to trusts and patients that it is safe to return to pre-pandemic levels of elective orthopaedic surgery.
